# Understanding women's uptake and adherence in Option B+ for prevention of mother-to-child HIV transmission in Papua, Indonesia: A qualitative study

**DOI:** 10.1371/journal.pone.0198329

**Published:** 2018-06-18

**Authors:** Christina Lumbantoruan, Michelle Kermode, Aloisius Giyai, Agnes Ang, Margaret Kelaher

**Affiliations:** 1 Centre for Health Policy, University of Melbourne, Melbourne, Victoria, Australia; 2 Nossal Institute for Global Health, University of Melbourne, Melbourne, Victoria, Australia; 3 Provincial Health Office, Papua Provincial Health Office, Jayapura, Papua, Indonesia; Johns Hopkins School of Public Health, UNITED STATES

## Abstract

**Background:**

Despite a more proactive approach to reducing new HIV infections in infants through lifelong treatment (Option B+ policy) for infected pregnant women, prevention of mother-to-child transmission of HIV (PMTCT) has not been fully effective in Papua, Indonesia. Mother-to-child transmission (MTCT) is the second greatest risk factor for HIV infection in the community, and an elimination target of <1% MTCT has not yet been achieved. The purpose of this study was to improve understanding of the implementation of Option B+ for PMTCT in Papua through investigation of facilitators and barriers to women’s uptake and adherence to antiretroviral therapy (ART) in the program. This information is vital for improving program outcomes and success of program scale up in similar settings in Papua.

**Methods:**

In-depth interviews were conducted with 20 women and 20 PMTCT health workers at two main referral hospitals for PMTCT in Papua. Development of interview guides was informed by the socio-ecological framework. Qualitative data were managed with NVivo11 software and themes were analysed using template analysis. Factors influencing women’s uptake and adherence in Option B+ for PMTCT were identified through final analysis of key themes.

**Results:**

Factors that motivated PMTCT uptake and adherence were good quality post-test HIV counselling, belief in the efficacy of antiretroviral (ARV) attained through personal or peer experiences, and a partner who did not prevent women from seeking PMTCT care. Key barriers for PMTCT participation included doubts about ARV efficacy, particularly for asymptomatic women, unsupportive partners who actively prevented women from seeking treatment, and women’s concerns about community stigma and discrimination.

**Conclusions:**

Results suggest that PMTCT program success is determined by facilitators and barriers from across the spectrum of the socio-ecological model. While roll out of Option B+ as current national policy for pregnant women in Papua has improved detection and enrolment of HIV-positive women, health facilities need to address various existing and potential issues to ensure long-term adherence of women beyond the current PMTCT program, including during pregnancy, childbirth and breastfeeding.

## Introduction

In 2012, the World Health Organization (WHO) recommended Option B+ as a novel approach to eliminate mother-to-child transmission (MTCT) of HIV [1]. This approach requires routine HIV testing for all pregnant women and lifelong antiretroviral therapy (ART) for positive cases irrespective of HIV clinical status or CD4 count [2]. Based on WHO criteria, elimination of mother-to-child transmission (EMTCT) of HIV is achieved when there is less than 2% MTCT in non-breastfeeding populations or less than 5% in breastfeeding populations, and if per 100,000 live births there are no more than 50 new pediatric infections [3].

Papua is the largest province of Indonesia that comprises most of Western New Guinea, and is the homeland to over three million indigenous Papuans. To the east, it is bordered by the nation of Papua New Guinea (PNG) and by West Papua province to the west. Papua is internationally known or referred to as ‘West Papua’, especially among the free movement coalitions [4].

Papua is one of the least populated, but most geographically challenging regions, in Indonesia with limited health infrastructure development, resulting in poor health status of the Papuan people [5]. Antenatal coverage is the lowest in Indonesia with less than 25% of pregnant women completing four mandatory antenatal clinic (ANC) visits (K4) during pregnancy, [6] and the child mortality rate is the highest in the country with 115 deaths per 1,000 live births [7]. Moreover, it is the only region in Indonesia that has documented a generalised HIV epidemic at 2.3%, which is mainly transmitted through heterosexual contact, [8] with perinatal transmission the second main risk factor for HIV. With an estimated 3.6% of 87,230 pregnant women in Papua being HIV positive [9], the Indonesian government has committed to elimination of MTCT in Papua through adoption of Option B+ since 2012.

Prior to Option B+, PMTCT performance in Indonesia was suboptimal with 86 new HIV infections in 1,145 (7.5%) live births among HIV-positive women who received the intervention [9]. During this period, PMTCT implementation was limited because of stigma and discrimination, long distances to health facilities, and long waiting times [10–12]. After adoption of Option B+, little is known about the experiences of HIV-positive women attending the PMTCT program, and the policy impact of PMTCT uptake/adherence and MTCT prevalence in Papua and at the national level.

To address this lack of evidence, our study aimed to document the impact of Option B+ and women’s experiences in PMTCT through qualitative (Papua) and quantitative (national) study. This paper reports on results from the qualitative study about women’s experiences of Option B+ in Papua, Indonesia. The quantitative study that examined PMTCT uptake and adherence, and the MTCT rate with Option B+, is being conducted at a national level and will be completed by the end of 2018.

## Methods

### Study setting

The study was conducted at government health facilities providing PMTCT services in Jayapura city, Jayapura Hospital (urban) and Abepura Hospital (suburban). Both hospitals have implemented the PMTCT program since 2004 [13] and are PMTCT referral sites for satellite primary health centers (PHC) and public hospitals within and outside of Jayapura city. Jayapura Hospital has nine satellite facilities (six PHCs in Jayapura city, one PHC in Waropen District, and two PHCs in Keerom District) and Abepura Hospital has eight satellite facilities (five PHCs in Jayapura city, three hospitals in Puncak Jaya District, Yapen Island, and Keerom District) [14].

Similar to HIV programs for other populations, PMTCT is implemented at referral hospitals and satellite centres. For PMTCT purposes, all pregnant women newly diagnosed with HIV at satellite centres are referred to referral hospitals for post-test HIV counselling and PMTCT initiation. Women from satellite centres have the option to continue the PMTCT program at the original satellite centre or at a referral site. Exceptions apply to pregnant women from other districts, such as Waropen, Keerom, Puncak Jaya and Yapen, requiring air transportation to reach Jayapura city. In this case, satellite centres initiate and monitor women in PMTCT programs through coordination with referral hospitals and report data to their referral hospital on a monthly basis [15].

PMTCT program implementation at both referral hospitals involves antenatal clinic (ANC), maternal ward, laboratory, and Care Support and Treatment (CST) clinic. According to the national PMTCT guideline [15], every pregnant woman visiting an ANC and every woman in a maternal ward with an unknown or negative HIV status should be tested for HIV. Upon testing positive, women are referred to the CST clinic for HIV post-test counselling and PMTCT initiation and monitoring, while the ANC focuses on pregnancy care. However, while national guidelines outline ideal procedures, field implementation may vary based on resource availability at implementing health facilities.

### Study population and sampling

This study investigated factors influencing women’s uptake and adherence in Option B+ based on experiences of women and health workers in the PMTCT program. Hence, participants in this qualitative study were pregnant and postpartum HIV-positive women with live birth who attended the ANC at Jayapura Hospital and Abepura Hospital, as well as health workers who provided PMTCT services at both health facilities.

We defined PMTCT uptake and adherence by combining the WHO definition of MTCT, the WHO guideline for Option B+ PMTCT, and the national guideline for PMTCT. WHO defined MTCT as transmission of HIV from mother-to-child during pregnancy, childbirth and breastfeeding [16]. Option B+ PMTCT requires HIV-positive women to start lifetime ART as soon as diagnosed, while the infant receives prophylaxis for six weeks regardless of infant feeding method [17]. In this study, we defined PMTCT uptake as HIV-positive women starting ART on the day HIV diagnosis is informed through post-test HIV counselling [18, 19]. Meanwhile, adherence was defined as 100% ART compliance based on pill count and confirmed by clinical observation during pregnancy, childbirth and breastfeeding. Women who missed appointments and were therefore without ART for some time during the MTCT period were classified as non-adherent.

Women participants were purposively selected to meet a target of 20 women representing two categories of participants in Option B+. These two categories were: 1). Women who accepted PMTCT, but were non-adherent to the program (n = 10), and 2). Women who accepted PMTCT and were retained in the program (n = 10). A minimum sample of 10 women per category was established to achieve data saturation [20], which was detected by CL during the last few interviews, and confirmed during data analysis.

In the original design, we included a third category of women who declined the offer of PMTCT, but returned for pregnancy or infant care, including immunisation or infant HIV diagnosis, at 18 months. However, we removed this category because health workers claimed that all pregnant women had accepted the PMTCT offer at both hospitals since Option B+ was implemented.

The first category was also revised from ‘women who accepted PMTCT, but discontinued the program’ to ‘women who accepted the PMTCT, but were non-adherent to the program’ due to the strategy implemented by both health facilities to bring women who missed their appointments back into the program through follow up phone calls or coordination with ANCs and maternal wards.

All health workers providing PMTCT services for at least 12 months (n = 20) at ANC, CST, and maternal wards of both hospitals were included in the study. Health workers interviewed consisted of HIV counsellor/head of ANC (n = 2), HIV counsellor/head of maternal ward (n = 2), CST GP/specialist (n = 2), CST HIV counsellor nurse/midwife (n = 2), CST nurse (n = 3), CST pharmacist (n = 4), CST laboratory/logistics (n = 2), and CST data and administration (n = 3).

### Selection and recruitment of participants

As part of the recruitment process, CL conducted preliminary visits to both hospitals to discuss the women they encountered and women required for the study. CL also visited peer support groups for women, Yayasan Harapan Ibu (YHI), and met with a peers in Jayapura city to identify HIV-positive pregnant and lactating women who were not in PMTCT, but without success.

We therefore continued with recruitment of respondents at both hospitals in collaboration with health workers. Prior to data collection, CL conducted an information session with health workers at each hospital to explain the purpose and approach of the study. Health worker involvement in the study was limited to assessing each visiting woman against study criteria, with study introduction and recruitment of women conducted by CL.

Each woman referred to CL received written and verbal study information in Indonesian, the national language spoken in Papua, by CL. A written consent form was signed by women who agreed to participate. Acceptance rate was 100% (20/20). All participants, including health workers, were informed of the right not to participate and to withdraw from the study at any time.

Women who agreed to participate were asked about their time and location preference for interviews. All women preferred to be interviewed on the same day at the hospital while waiting for their appointment, other than one woman who requested to return for an interview the following day. Length of interviews ranged from 15 to 45 minutes with an average of 29 minutes, excluding icebreaking conversation that took around 5 minutes per interview.

Health worker participants were individually approached by CL and were informed about the voluntary nature of the study. All health workers (20/20) participated voluntarily in interviews. As preferred by health workers, interviews were conducted at the hospitals after working hours or during less busy hours. Duration of interviews with health workers was between 16 and 60 minutes with an average of 38 minutes.

### Data collection

CL, who was experienced in qualitative interviewing techniques, conducted all one on one in-depth interviews from August to December 2016 in a private room at the care support and treatment (CST) clinic. In-depth interviews with HIV-positive women and health workers were guided by a conceptual framework, explained in the following section. The framework was developed based on study objectives and key findings from literature on HIV and PMTCT in Indonesia and other low and middle income countries.

Interview guides were developed in English and translated into Indonesian before being piloted at YHI, the local peer support NGO group and at Abepura Hospital in Jayapura city. All interviews were audio-recorded with consent from participants. Sociodemographic data was gathered from both participant groups. Women participants were asked what motivated them to participate in the PMTCT program, what helped them to remain in the program, and what prevented them from remaining in the program. Health workers were asked about work responsibilities, management of pregnant women newly diagnosed with HIV including pre and post HIV test counselling, privacy and confidentiality, and factors facilitating and inhibiting women’s acceptance and continuation in the program. Interviews also explored participant recommendations for improvements to PMTCT uptake and adherence. The interviewer used the conceptual framework as a guideline to explore key themes not mentioned by participants but found in literature reviews or identified by the interviewer during her previous work with the HIV program in Papua.

### Ethics approval and consent to participate

Ethical clearance for the study was obtained from the Health Sciences Humans Ethics Sub-Committees (HESC) of the University of Melbourne, Australia, as well as written research approvals from the Indonesian Ministry of Home Affairs, Papua Provincial Health Office, Jayapura Hospital, and Abepura Hospital. Participation in the study was voluntary and participants were free to withdraw at any stage. Study information was provided in writing and verbal form, and written consent was obtained. Confidentiality and anonymity of participants was maintained by de-identification of personal data and non-inclusion of information that might identify individual participants.

### Conceptual framework

**Fig 1:**
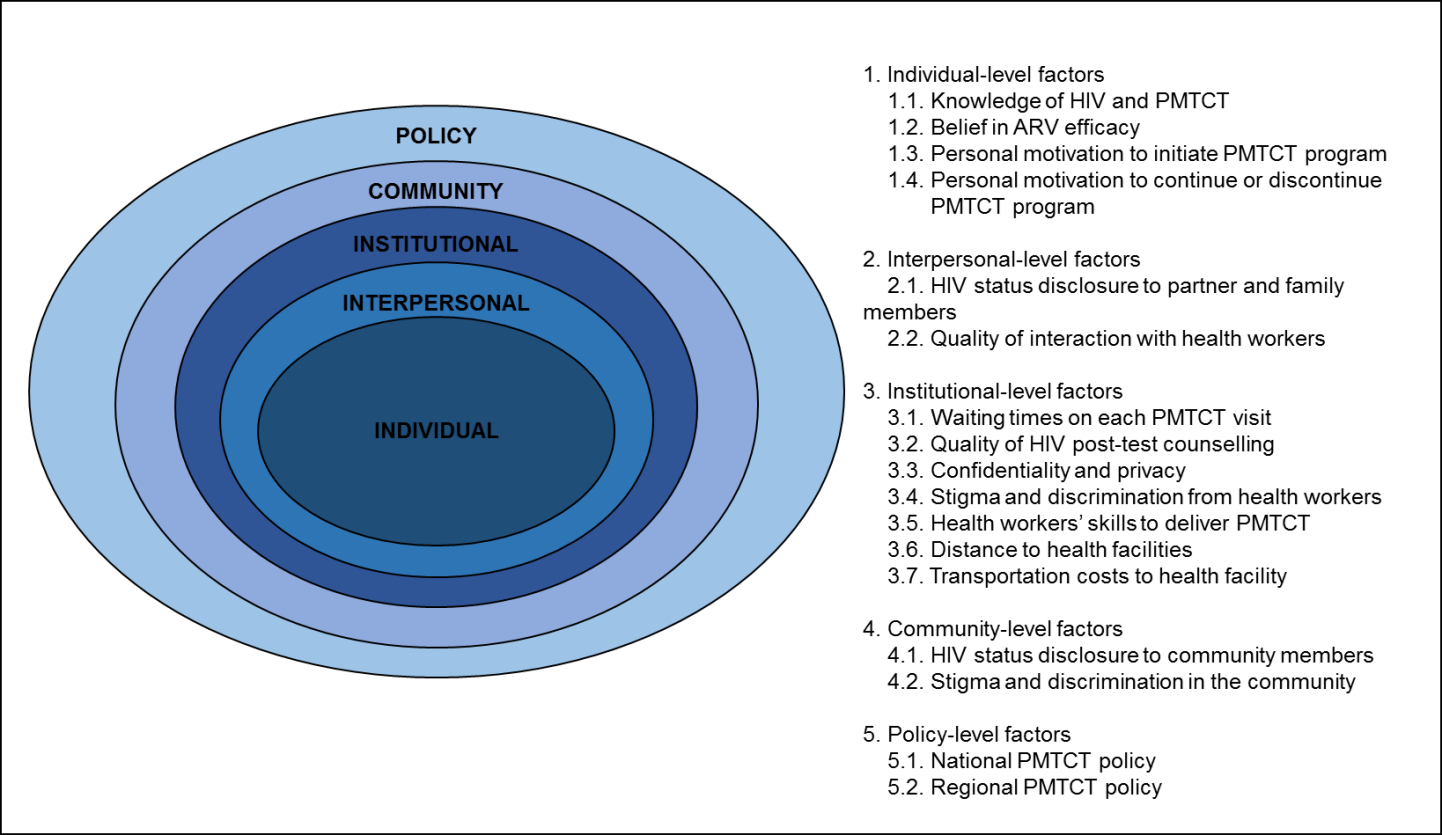
The modified socio-ecological framework consists of five levels of *a priori* themes. PMTCT = Prevention of Mother-to-Child Transmission of HIV.

An adjusted socio-ecological framework [21] informed development of *a priori* themes for qualitative analysis of interview transcripts. This model consists of five levels of influence: individual, interpersonal, institutional, community and policy factors, as presented in Fig 1. Individual-level factors were defined as a woman’s knowledge of HIV and the PMTCT program, reasons for HIV testing, belief in ARV efficacy, personal motivation to initiate engagement with the PMTCT program, and personal motivation to continue or discontinue with the PMTCT program. Interpersonal-level factors included willingness to disclose HIV status to partner and family members (or not), and quality of interactions with PMTCT health workers. Institutional-level factors focused on the health care system supporting PMTCT at health facilities, including waiting times, quality of HIV post-test counselling, confidentiality and privacy, stigma and discrimination from health workers, distance to health facilities, and cost of transportation to health facilities. Community-level factors included HIV status disclosure to non-family members, and stigma and discrimination in the community. At the policy level, identified factors were national policy and the hospital policy for PMTCT that influence the nature and quality of program delivery at health facilities.

### Data analysis

CL initially transcribed interviews in Indonesian language and then translated into English. Data was managed using NVivo11 software followed by application of template analysis, a form of thematic analysis that utilises hierarchical coding with adjustable structures for analysing qualitative data [22]. CL conducted the first coding using *a priori* themes established by the research team prior to data collection, and added new emerging themes. Themes were then discussed with MR and ME for validation and finalisation. The research team followed eight steps for conducting template analysis:

Step 1. Development of *a priori* themes based on literature related to the PMTCT program in Indonesia and other low and middle income countries, as shown in Fig 1.
Step 2. Data familiarisation by reading interview transcripts.
Step 3. Preliminary data coding by highlighting parts of transcripts relevant to *a priori* themes and new information relevant to research questions.
Step 4. Refinement of *a priori* themes to integrate new emerging themes.
Step 5. Organisation of themes into groups and establishment of hierarchical and lateral relationships between groups.
Step 6. Development of an initial coding template after coding five interviews that contained the greatest variation in responses.
Step 7. Application of the initial coding template, and revision after coding of another five interviews.
Step 8. Finalisation of the coding template and its application on the full data set.

## Results

### Participant characteristics

Data from 40 interviews, 20 HIV-positive women (10 women who were non-adherent, 10 women who were 100% adherent) and 20 health workers (health workers providing PMTCT service at both hospitals for at least one year), were analysed. Women in the non-adherent category stopped taking ART between seven days and over three months with an average of 49.7 missing days. All HIV positive women participants were indigenous Papuans, but ethnicity varied with the majority belonging to ethnic groups residing outside of Jayapura (Table 1). Health workers were predominantly from other parts of Indonesia and had migrated to Papua during childhood or for work placement, but nevertheless identified themselves as Papuans. The majority of HIV-positive women were Christian, while health workers were predominantly Muslim. Ninety per cent of women described themselves as either married or living with their partners. A majority of health workers were married. Most women were high-school graduates, with only four having attended college or university.

**Table 1:** Sociodemographic characteristics of participants (n = 40).

Characteristics	Frequency	
Participants	HIV-positive women	Health workers
Age		
Younger than 25 years	8	0
26–30 years	6	3
31–40 years	6	13
Over 40 years	0	4
Gender		
Male	0	4
Female	20	16
Marital status		
Married	5	16
Living with partner	13	1
Divorced/Separated	0	1
Single	2	2
Number of Children		
No children	0	4
1–3 children	18	16
More than 3 children	2	0
Education level		
Elementary	3	0
Secondary	2	0
High school	11	0
College/University	4	20
Religion		
Christian	18	9
Muslim	2	11
Ethnicity		
Papuan from Jayapura city/district	3	2
Papuan from outside of Jayapura	17	2
Non-Papuan	0	16
HIV status		
Partner tested and both HIV positive	9	N/A
Partner tested but HIV negative	4	N/A
Partner not tested for HIV	7	N/A

N/A = Not Available

Findings are presented based on final themes for each level of the socio-ecological framework that influenced PMTCT treatment uptake and adherence by HIV-positive women in Papua (S1 File).

### Facilitators and barriers to Option B+ uptake and adherence

#### 1. Individual-level factors

**HIV counselling and PMTCT treatment uptake:** In both adherent and non-adherent groups, contrary to health worker information, some women claimed they had no idea their blood was tested for HIV. As explained by a health worker:

“HIV testing in Option B+ was offered as a part of regular antenatal examination package that does not require a separate consent form. Hence, provision of pre-test HIV counselling which was combined with other health information might not be obvious to some women” (HealthWorker#18, 35 years)

However, women who reported not receiving pre-test HIV counselling did not object to this oversight and said that post-test HIV counselling improved their understanding of the disease and its treatment. Most women said they accepted PMTCT treatment as a result of post-test HIV counselling. As explained by a woman who was tested for HIV around the time of childbirth:

“I did not know I was tested for HIV. Later after the result came out, I [first] heard about it at the maternity ward and then I was brought here [CST clinic], I was really shocked…I cried…cried…but they encouraged me and advised that if I take the medicine regularly, it means I can be healthy like other normal people. So, I started the treatment” (Woman#3, 30 years)

**Belief in ARV efficacy and PMTCT adherence:** While good knowledge of HIV and PMTCT helped women in our study accept the program, it did not necessarily guarantee adherence to medication or the program. In this cohort, belief in the efficacy of ARV was an important factor promoting adherence to the program. Some women’s beliefs about treatment efficacy were reinforced by personal experience or witnessing positive health outcomes of peers after taking ARVs. For others, trusted knowledge was provided by health workers who stated that without ARV their health would deteriorate, advancing from asymptomatic to symptomatic HIV. In this study, non-adherent women either had doubts about the efficacy of ARVs (4/10) or experienced prolonged side effects (1/10). One woman who stopped her medication explained:

“I have not experienced any benefit of the medication. Each morning I wake up feeling dizzy …very dizzy…I had been taking the medicines for two months and the dizziness did not disappear. So, I don’t feel like returning for the medicines” (Woman#4, 18 years)

Another woman stopped her treatment for three months:

“I find it hard to believe that ARVs would help prevent its transmission to my infant. It is incurable; there really is no hope for us.” (Woman#13, 34 years)

#### 2. Interpersonal-level factors

**HIV status disclosure and partner support**: The majority of women in this study (16/20) had disclosed their HIV status to their partners and requested they get tested for HIV. Sero-discordant and HIV positive partners were reported to be more supportive than partners who refused to get tested. The latter could be mentally or physically abusive, and prevented women from adhering to treatment. The presence of domestic violence (3/10) before and/or after HIV status disclosure became a main reason for PMTCT non-adherence reported by women. As stated by one woman:

“My partner was still upset because of my illness. I did not drink my medicines for two weeks because he threw away all of my medicines when I was not home. When I asked him, he said ‘I was collecting rubbish in the house’, so he threw them away. I could not return to the health facility to get more medicines because he wouldn’t give me any money for transport” (Woman#16, 23 years)

However, having a supportive partner did not guarantee women’s adherence to the program. As a non-adherent woman explained:

“My partner is very supportive. He is the one to remind me to take my medicines and bring me to the hospital to top up my drugs. He would persuade me to drink my medicine when I didn’t feel like it” (Woman#4, 18 years)

#### 3. Institutional-level factors

Health workers explained the increasing number of patients they managed at CST clinics because HIV+ pregnant women no longer stopped ART after childbirth/breastfeeding as occurred pre- Option B+. They explained major infrastructure and human resources changes were less likely to occur in the short-term, so the PMTCT program was adjusted to meet available resources. Consequently, there was a reduction in quality of care, such as long wait times and lack of privacy, but women and health workers rarely identified these as barriers to PMTCT uptake and adherence. Frequently mentioned facilitators of program adherence were respect for confidentiality and stigma-free care from health workers.

**Wait times:** All women (20/20) stated wait times were long, between two to five hours, depending on the number of child patients on the day. Nearly all women (19/20) understood and accepted this situation, but a few women (2/20) hoped wait time could be reduced in the future. Only one woman identified long wait time as a reason for non-adherence:

“Too long. Wasting time. Also, I waited for so long [from morning until past lunch time] until I felt very hungry. All these make me reluctant to comeback” (Woman#13, 34 years)

**Privacy and confidentiality:** Both health workers and women commented on respect for confidentiality during post-test HIV counselling. The majority of women (19/20) received the test result in person, in a private counselling room, uninterrupted, and unheard by other patients or health workers at the clinic. There was an exceptional case involving a woman who received her HIV test result at a satellite health facility in the presence of a second health worker who stayed in the room during the counselling process. While this made the woman feel uneasy, it did not deter her from attending the PMTCT program.

Health workers explained strategies they use to maintain confidentiality of women’s HIV status outside of the health facility:

“Only health workers in the CST clinic know their HIV status. We explained to them that all of us in the clinic need to know because each of us deals with them for different reasons, such as blood testing, drug distribution, adherence monitoring, etc. But we promised them that we would do everything we can to make sure all information about them remain in this [CST clinic] room” (HealthWorker#1, 30 years)

“In the community setting, it is up to them to talk [to us] or not. There are patients who do not talk to us outside of this CST clinic. But it is okay. It is their right. We would not initiate a conversation with them unless they started. This to prevent people from assuming their HIV status because people know we are working in HIV clinic” (HealthWorker#2, 40 years)

**Stigma and discrimination at health facility:** Discrimination was not observed during field observations at both health facilities. There was no excessive use of precautions, including masks and gloves, in executing routine tasks or when meeting HIV+ women. Women participants also claimed they were not treated differently (20/20) after HIV diagnosis. No participant reported discontinuing treatment due to discrimination at the health facilities.

**Women-health worker relationships:** The relationship between health workers and women seemed to be satisfactory as all women (20/20) described health workers as either friendly or kind, while health workers felt ‘*kasihan*’, or sympathy, for women and their children. A minority of women (2/20) mentioned health workers becoming angry with them when they missed their doses, but they believed it was ‘a sign of caring’, rather than dislike. One woman, however, preferred health workers explaining things without being angry.

#### 4. Community-level factors

**Stigma and discrimination in the community:** Perceived stigma and consequent discrimination in the community was seen by women as an important barrier to continued participation in the PMTCT program. Hence, a large proportion of women (16/20) sought treatment at a health facility outside of their neighborhood to avoid detection of HIV diagnosis by family members or friends. For this group, this meant travelling between 45 and 60 minutes using public transportation (n = 7) or continuing PMTCT treatment at referral hospitals instead of returning to nearby satellite PHCs (n = 9). Of 10 women who were adherent to PMTCT treatment, only one woman lived less than 30 minutes from the health facility.

Some women preferred to increase the distance from home to health facility, as explained by one woman:

“I decided to move out of our previous neighborhood since diagnosed with HIV. Now we live far away from people we know. There is another hospital near our current house but I have family members working there. I am afraid they will find out. It is safer if I come here” (Woman#10, 30 years)

A woman who lived near the health facility attributed fear of discrimination as a reason for discontinuing ART:

“I am afraid of being found out by my relatives and friends if returning to this clinic. I’ve got friends and family living around here. Every time I come, there are many people seeing me coming. Only HIV positive people come to this clinic for medication. So, I feel ashamed. All this makes me reluctant to come back. I stopped my treatment for this reason” (Woman#13, 34 years)

All adherent and non-adherent women in this study experienced increased transportation costs as increased travel distance was a less significant issue compared to anticipated stigma and discrimination. However, two women had missed appointments due to lack of money for transportation.

#### 5. Policy-level factors

**Regional health insurance:** A main facilitator for women’s uptake and adherence in the PMCTC program was ‘free of charge’ HIV services funded by the government through the Papua Health Scheme ‘*Kartu Papua Sehat/KPS*’ implemented by the Papua government in 2014. This scheme provides free health services, including medicines and laboratory testing, for underprivileged indigenous Papuans. As stated by health workers:

“Majority of Papuan women sought HIV treatment using KPS. They do not have any health insurance so we registered them for KPS” (HealthWorker#2, 40 years)

“Everything is free. HIV-positive people receive special treatment. Free medicine. Free laboratory testing” (HealthWorker#5, 25 years)

While benefiting the women, health workers mentioned a disadvantage of KPS in terms of difficulty recruiting additional health workers at CST clinics due to absence of incentives:

“Many health workers are unwilling to take their post at the Voluntary Counseling and Testing/VCT clinic because there is no incentive [KPS does not contain any incentives for health workers. Other health insurance schemes including The Indonesian National Health Insurance System ‘*Badan Penyelenggara Jaminan Sosial/BPJS*’ provides monthly incentives for health workers based on the number of patients treated] to claim. I say, only people with a serving heart work here. We tried to request for additional workers because we need more forces, but without result because the appointed health workers never showed up” (HealthWorker#4, 28 years)

## Discussion

This qualitative study conducted at two PMTCT referral hospitals identified several motivators and barriers to women’s uptake and adherence in Option B+ that are comparable with findings from previous studies in resource-limited settings [23–26]. Factors motivating uptake and continuation in the program were a constellation of individual, interpersonal, institutional, and policy factors. Factors associated with increased uptake and adherence included good quality post-test HIV counselling, belief in the efficacy of ARVs to prevent transmission and improve health, confidentiality of HIV status, absence of stigma and discrimination at health facilities, positive women-health worker relationships, and free HIV services [25–32].

Our findings are consistent with previous research regarding the importance of post-test HIV counselling in helping women understand the disease and consequently promote participation in PMTCT programs [24, 27, 31–32]. Similar to a study in rural Tanzania, we found that PMTCT knowledge alone did not ensure women’s adherence to the program [33], which is in contrast to findings from Malawi and Uganda [34]. In our study women who were adherent to the program not only had good knowledge of ARV and PMTCT, but they also believed in the efficacy of ARVs to improve health status and prevent HIV transmission. Though the term ‘belief in ARV efficacy’ was not specifically mentioned, our findings are similar to other studies that reported desire for personal and infant health as a facilitator of PMTCT adherence [18].

Women participants in our study enrolled and remained in the program as a result of HIV post-test counselling that was understandable and provided them time to think. Effective communication at post-HIV test counselling and positive health worker-women relationships were also found to be important for adherence in the PMTCT program in other studies [25, 35]. This is different to findings of a study conducted in the highland of Papua, Indonesia, where migrant health workers were a barrier to PMTCT uptake and adherence [36]. The more positive outcomes in this study may relate to health workers’ self-identifying as Papuans, rather than as migrant workers, thus having better understanding of Papuan culture. Also, as explained by a health worker, the national government’s health policy for implementation of Option B+ for all pregnant women in Papua was accompanied by a range of training that significantly improved health worker knowledge and skills to effectively deliver the PMTCT program, including reduction of stigma and discrimination. Women seeking treatment in Jayapura city did not experience noticeable discriminatory behaviours on the part of health workers.

The women in our study did not necessarily require a partner who actively supported their treatment in order to remain in the program, unlike findings in other studies [23, 25, 37, 38]. A majority of women continued their treatment due to personal belief in ARV efficacy even without active encouragement from their partner. However, consistent with findings of other studies, partner support is important to retain women who are financially dependent on the partner [25, 26, 37, 39, 40].

The main barriers to adherence were disbelief in ARV efficacy to prevent HIV transmission from mother to child, unsupportive partners, and fear of stigma and discrimination from community members [23, 38, 41–43]. Women’s doubts regarding ARV efficacy were commonly reported as a main challenge to adherence in PMTCT for asymptomatic participants, as documented in several studies in East Africa [19, 33, 44], as they do not experience a tangible benefit from treatment. Similar to experiences of women in our study, some women who were not adherent felt healthy without treatment and others did not believe that ARVs would make a difference for them and their infants [19, 33, 44].

A review of the PMTCT program pointed out the role of stigma and discrimination at the community level as a barrier to PMTCT participation [45]. In this study, a majority of women sought treatment far outside their neighborhoods due to fear of HIV status exposure in the local community. This unnecessarily added to the burden of the PMTCT program for women by increasing travel time and cost of transportation. Though both consequences were not reported as a major barrier to PMTCT adherence by women participants, it did become a reason why financially-dependent women missed appointments.

The Indonesian government aims to eliminate PMTCT by reducing HIV vertical transmission to less than 1% and by implementing the PMTCT program nationwide by 2019 [46]. Findings of this study highlight changes that can be made to improve women’s uptake and adherence in the PMTCT program. Various studies have suggested a need to improve HIV post-test and follow up counselling quality [47, 48] to increase women’s awareness and knowledge of HIV and PMTCT. Other studies suggested a need to engage male partners in PMTCT [23, 49] to increase mutual communication and create support systems needed by women. In this study, this was particularly relevant for women who missed their appointments due to unsupportive partners. To address anticipated stigma and discrimination in the community, various interventions could be considered including reduction of stigma in the community through continuous campaign, prevention of internalisation of perceived community stigma, and targeting the development process of perceived stigma [50]. Lastly, even though women in our study did not identify the following factors as barriers to PMTCT uptake and adherence, quality of care could be much improved through recruitment of adequate numbers of health workers, improvement of privacy and reduction of wait times [25, 51].

**Study limitations.** In this study, interviews explored themes beyond the *a priori* themes and were enriched through use of field observations that we consider a strength of the study. Both health facilities selected for this study are located in urban and sub-urban Jayapura, but the majority of women participants were from outside of Jayapura. This contributed to richness of information we collected. However, we also realise that the information collected is limited to reported experiences of participants in the PMTCT program at referral hospitals, with a glimpse of PMTCT implementation at satellite health facilities provided by referred women as we did not conduct physical visits to satellite centres. Moreover, participants in this study were women attending referral hospitals and may not necessarily reflect the situation of women at satellite facilities or more remote areas of Papua. Therefore, caution needs to be applied when interpreting and generalising study findings to other districts in Papua and Indonesia by taking into account possible variations influencing factors at all levels.

## Conclusions

Our study argues the clear importance of motivating factors that outweigh barriers to PMTCT uptake and adherence at five levels of the socio-ecological framework. The roll out of Option B+ as policy for pregnant women in Papua, which means inclusion of HIV testing as a routine part of pregnancy screening, has improved identification of HIV-positive women and their enrolment in the program. Further strengthening of the PMTCT program is necessary to ensure continuous enrolment of new cases while maintaining adherence of women in HIV care by addressing barriers or potential inhibitors to long-term treatment. These include availability of strategies to identify and counsel women with doubts regarding ARV efficacy early in the program, establishment of support for women in need, a continuous campaign to reduce stigma and discrimination at the community level, availability of adequate human resources, reduction of long waiting times, and increased privacy during return visits.

## Supporting information

S1 File Final themes of motivators, barriers and recommendations related to PMTCT uptake and adherence in Papua, Indonesia. (DOCX)

S2 File Interview guide for women in English. (DOCX)

S3 File Interview guide for women in Indonesian. (DOCX)

S4 File Interview guide for health worker in English. (DOCX)

S5 File Interview guide for health worker in Indonesian. (DOCX)
