# Supplementary material for: Understanding women's uptake and adherence in Option B+ for prevention of mother-to-child HIV transmission in Papua, Indonesia: A qualitative study
Source: PLoS One. 2018 Jun 18;13(6):e0198329. doi: 10.1371/journal.pone.0198329 (PMC6005458; doi:10.1371/journal.pone.0198329)
Supplement: S1 File — (DOCX) [file pone.0198329.s001.docx]

### S1 text. Final themes of motivators and barriers to PMTCT uptake and adherence in Papua, Indonesia

| **Facilitators** | **Barriers** |
| --- | --- |
| **1. Individual Level** |  |
| - 1. Knowledge of HIV and PMTCT | - 1. Knowledge of HIV and PMTCT |
| - - 1. Source of knowledge | 1.1.1. Source of knowledge |
| - - - 1. HIV post-test counsellor |  |
| - - - 1. Peers who are on HIV treatment | 1.1.1.1. Peers who are not on HIV treatment |
| - - - 1. Personal search that supports existing knowledge | 1.1.1.2. Personal search that questions existing knowledge |
| - - 1. Accuracy of knowledge | 1.1.2. Accuracy of knowledge |
| - - - 1. Correct knowledge | 1.1.2.1. Correct but incomplete knowledge |
| - - 1. Belief in the knowledge | 1.1.3. Disbelief in the knowledge |
| - 1. Belief in ARV efficacy | 1.2. Disbelief in ARV efficacy |
| 1.2.1. Personal experience | 1.2.1. Personal experience |
| 1.2.1.1. Improved health outcomes | 1.2.1.1. ARV side effects or worse health outcomes |
| 1.2.2. Close person’s experience | 1.2.2. Close person’s experience |
| 1.2.2.1. Improved health outcomes | 1.2.2.1. ARV side effects or worse health outcomes |
| 1.2.3 Other people’s experience | 1.2.3 Other people’s experience |
| 1.2.3.1. Improved health outcomes | 1.2.3.1. ARV side effects or worse health outcomes |
| 1.3. Personal motivation to initiate PMTCT program | 1.3. Personal motivation to initiate PMTCT program |
| 1.3.1. Expectation for a better health status | 1.3.1. Feeling guilty |
| 1.3.1.1. As a mother | 1.3.1.1. As the source of HIV in unborn infant |
| 1.3.1.1.1. To take care of her children | 1.3.2. Feeling obligated |
| 1.3.1.2. An HIV-negative infant |  |
| 1.4. Personal motivation to continue PMTCT program | 1.4. Personal motivation to discontinue PMTCT program |
| 1.4.1. Motivation to initiate treatment | 1.4.1. Weak motivation to initiate PMTCT to begin with |
| 1.4.1.1. Continuity of motivation | 1.4.2. Emerging challenges related to return visits |
| 1.4.1.2. Improvement of motivation | 1.4.2.1. Financial issues |
|  | 1.4.2.1.1. Transportation cost |
|  | 1.4.2.2. Conflicting priorities |
|  | 1.4.2.2.1. Working |
|  | 1.4.2.2.2. Mobility |
| **2. Interpersonal Level** |  |
| 2.1. HIV status disclosure to partner | 2.1. HIV status disclosure to partner |
| 2.1.1. Partner | 2.1.1. Partner |
| 2.1.1.1. Partner was informed and got tested | 2.1.1.1. Partner was informed and refused to get tested |
| 2.1.1.1.1. Both are HIV positive and on treatment |  |
| 2.1.1.1.2. Only the woman is positive, but partner supports her treatment | 2.1.1.1.1. Only the woman’s HIV status is known, and partner does not support her treatment including domestic violence related to HIV status |
| **3. Institutional Level** |  |
| 3.1. Patient waiting times for each PMTCT visit | 3.1. Patient waiting times for each PMTCT visit |
| 3.1.1. Understanding reasons for waiting times | 3.1.1. Waiting times could be reduced if wanted |
| 3.1.2. Acceptance of waiting times as a normal circumstance | 3.1.2. Waiting times resulted in negative consequences such as cranky children and hungry mothers |
| 3.2. Confidentiality and privacy | 3.2. Confidentiality and privacy |
| 3.2.1. HIV post-test counselling |  |
| 3.2.1.1. High confidentiality and privacy |  |
| 3.2.2. Return visits | 3.2.1. Return visits |
| 3.2.2.1. Lack of confidentiality and privacy, but patients are not aware of the other’s reasons to visit | 3.2.1.1. Lack of confidentiality and privacy that prevents a woman from returning |
| 3.3. Stigma and discrimination from health workers |  |
| 3.3.1. Health workers’ attitude to women |  |
| 3.3.1.1. Friendly and kind |  |
| 3.3.1.2. Not friendly but still doing their jobs |  |
| **4. Community Level** |  |
| 4.1. HIV status disclosure to family members | 4.1. HIV status disclosure to family members |
| 4.1.1. Parents | 4.1.1. Parents |
| 4.1.1.1. Parents are informed and support woman’s treatment | 4.1.1.1. Parents were not informed on HIV status |
| 4.1.1.2. Parents share responsibilities with woman to take care of her child/children |  |
| 4.1.2. Other family members | 4.1.2. Other family members |
| 4.1.2.1. HIV status is known to other family members, and they support her HIV treatment | 4.1.2.1. HIV status is unknown to other family members |
| 4.2. Stigma and discrimination in the community | 4.2. Stigma and discrimination in the community |
| 4.2.1. Overcome by the need to be on HIV treatment | 4.2.1. Fear of gossips and social alienation |
| **5. Policy Level** |  |
| 5.1. National PMTCT policy | 5.1. National PMTCT policy |
| 5.1.1. Papua as an HIV program priority | 5.1.1. Low ratio of health workers to patients |
| 5.1.1.1. All pregnant women should be tested for HIV | 5.1.1.1. Priority on HIV-positive women, curative rather than preventative |
| 5.1.1.2. All HIV-positive women should enrol in HIV treatment | 5.1.1.2. The importance of adherence to prevent drug resistance |
| 5.1.1.3. Free medication |  |
| 5.2. Regional PMTCT policy | 5.2. Regional PMTCT policy |
| 5.2.1. Flexibility in the use of health insurance | 5.2.1. Patients’ negative response to changes in health insurance |
| 5.2.1.1. Absence of ID card accepted | 5.2.2. Absence of incentive and difficulty in human resource recruitment |
|  |  |
