# Supplementary material for: Understanding women's uptake and adherence in Option B+ for prevention of mother-to-child HIV transmission in Papua, Indonesia: A qualitative study
Source: PLoS One. 2018 Jun 18;13(6):e0198329. doi: 10.1371/journal.pone.0198329 (PMC6005458; doi:10.1371/journal.pone.0198329)
Supplement: S2 File — (DOCX) [file pone.0198329.s002.docx]

#
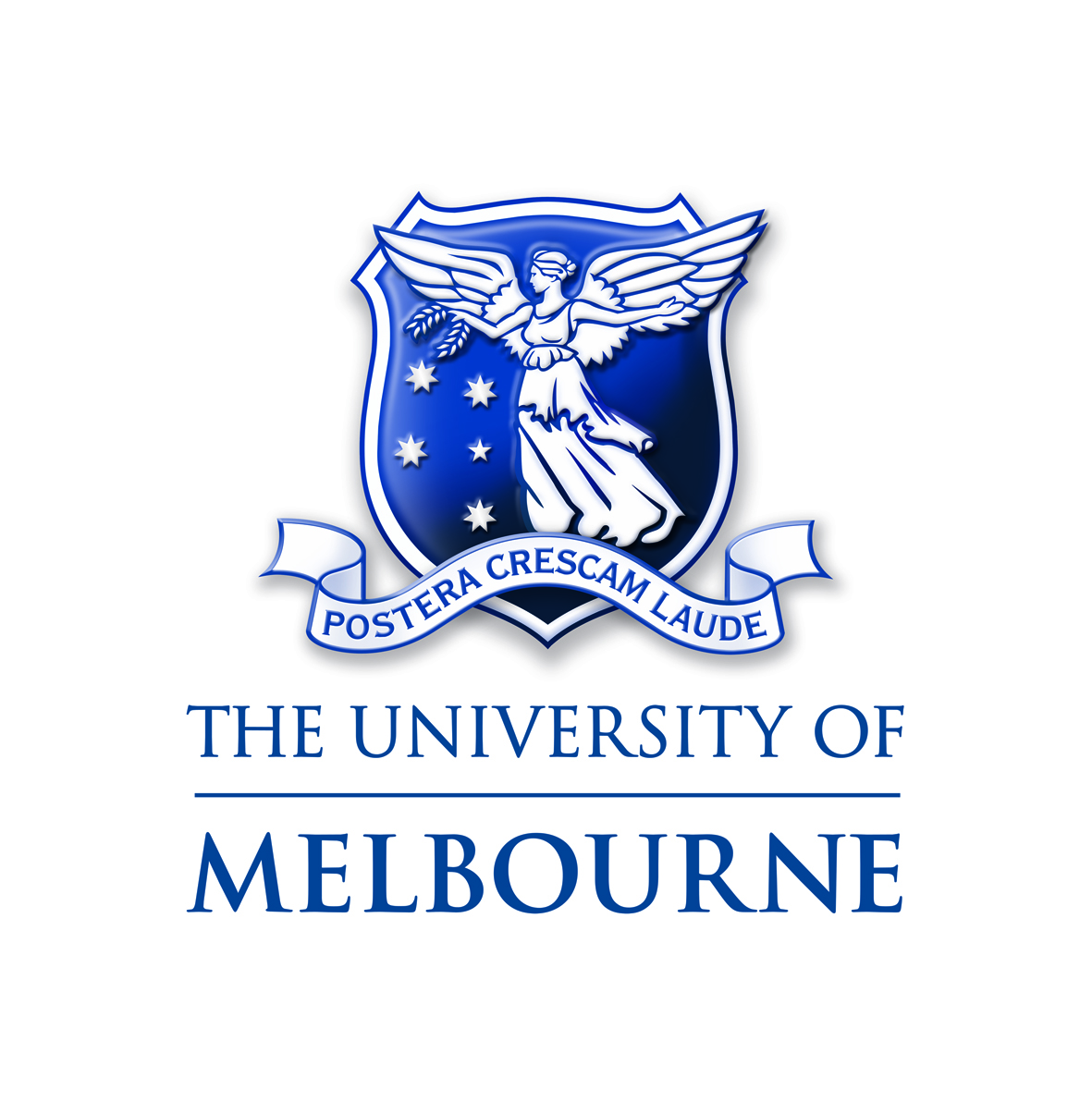
Interview Guide

## Centre for Health Policy

## ***Project:*** ***Understanding women’s uptake and adherence in Option B+ for prevention of mother-to-child HIV transmission in Papua, Indonesia: A qualitative study***

**Interview process:** The student researcher will conduct one on one in-depth interviews with women. The student researcher will explore each theme provided by participants and ask additional questions for missing or undetected themes before continuing to the next topic.

1. **Demographic information**
2. Age: _________ (years)
3. Ethnic background: _________________
4. Religion:__________________________
5. Educational background:
6. Did not complete primary school
7. Primary school
8. Junior high school
9. Senior high school
10. College/University
11. Occupation:
12. Housewife
13. Casual worker
14. Private business
15. Private sector
16. Civil servant
17. Marital status:
18. Single
19. Married
20. Divorced
21. Widow
22. Number of biological children:

_______ (person/s)

1. ANC visits for previous pregnancy(ies)

___________________________________

1. Status of last pregnancy:______(weeks) or age of the last child_______(months)
2. Distance from home to the health facility:________(hours)
3. Transportation cost to health facility: ____________(Indonesian rupiah)/trip
4. Modes of transportation involved: ___________________________________

| **III. Interview topics** | **Themes to explore** |
| --- | --- |
| **Topic 1: HIV testing experience during ANC visits.**  Question: “*Can you tell me about your HIV testing experience during ANC visits?”* | - Knowledge of HIV - Knowledge of PMTCT - Health workers-patient interaction - Reasons for HIV testing acceptance - Previous history of HIV testing |
| **Topic 2: Experience in PMTCT enrolment.**  Question: “*How did you learn about the result of your HIV test?”* | - Waiting time for the test result - Counselling duration - Confidentiality and privacy - Reasons for enrolment/not enrolment in PMTCT program - Health workers’ attitude toward the woman before and after tested positive for HIV |
| **Topic 3: Experience in PMTCT retention.**  Question: “*What drives you to (not) remain in the program?”* | - Facilitators and barriers to retention in PMTCT program: - Transportation cost - Distance to health facility - Stigma and discrimination - Dietary requirements - Belief in PMTCT efficacy |
| **Topic 4: Suggestions to improve PMTCT program.**  Question: “*How do you think the current PMTCT program could be improved?”* | - PMTCT strategies for pregnant women under various circumstances: - Social constraints (stigma and discrimination) - Geographical constraints - Financial constraints - Other issues mentioned by the woman |
| **Topic 6: Closing**  Question: *“Is there anything more you would like to add?”* |  |
