# Supplementary material for: Understanding women's uptake and adherence in Option B+ for prevention of mother-to-child HIV transmission in Papua, Indonesia: A qualitative study
Source: PLoS One. 2018 Jun 18;13(6):e0198329. doi: 10.1371/journal.pone.0198329 (PMC6005458; doi:10.1371/journal.pone.0198329)
Supplement: S3 File — (DOCX) [file pone.0198329.s003.docx]

#
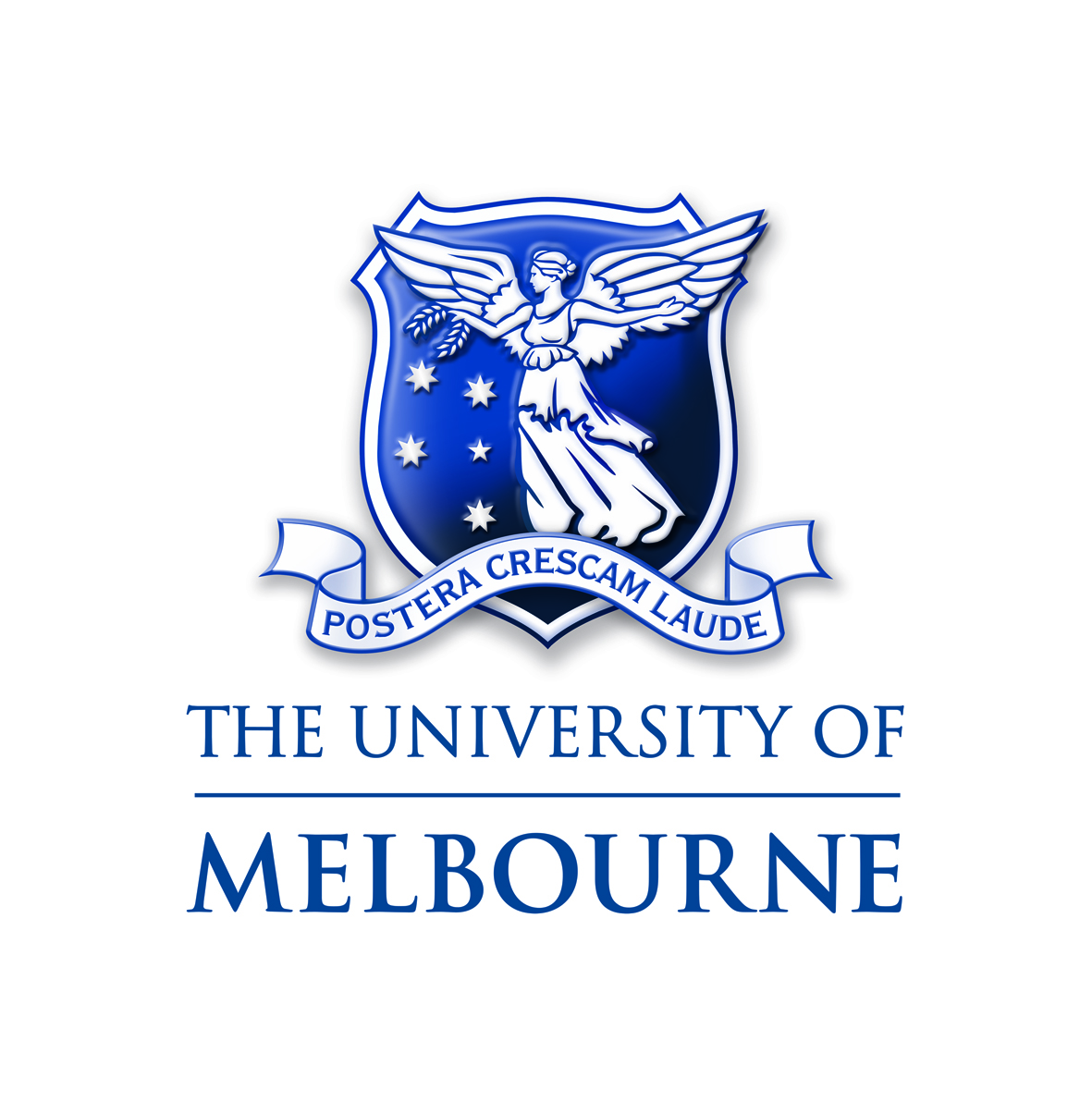
Panduan Interview

## Pusat Kebijakan Kesehatan

## ***Proyek: Memahami penerimaan dan kepatuhan wanita dalam Opsi B+ untuk pencegahan transmisi HIV dari ibu ke anak di Papua, Indonesia: Sebuah studi kualitatif***

**Proses Interview:** Mahasiswa peneliti akan melakukan wawancara mendalam satu persatu dengan perempuan. Mahasiswa peneliti akan menggali setiap tema yang disebutkan oleh peserta dan mengajukan pertanyaan tambahan untuk tema yang hilang atau tidak disebutkan sebelum melanjutkan ke topik berikutnya.

1. **Informasi demografis**
2. Usia: _________ (tahun)
3. Latarbelakang etnis: _________________
4. Agama:__________________________
5. Latarbelakang pendidikan:
6. Tidak menyelesaikan sekolah dasar
7. Sekolah dasar
8. Sekolah menengah pertama
9. Sekolah menengah atas
10. Universitas
11. Pekerjaan:
12. Ibu rumah tangga
13. Pekerja sambilan
14. Usaha pribadi
15. Sektor swasta
16. Pegawai negeri sipil
17. Status pernikahan:
18. Tidak menikah
19. Menikah
20. Cerai
21. Janda
22. Jumlah anak biologis:

_______ (orang)

1. Kunjungan KIA pada kehamilan terdahulu

___________________________________

1. Status kehamilan:______(minggu) atau usia anak terakhir_______(bulan)
2. Jarak dari rumah ke fasilitas kesehatan:________(jam)
3. Biaya transportasi ke fasilitas kesehatan: ____________(rupiah)/perjalanan
4. Jenis transportasi yang dibutuhkan: ___________________________________

| **III. Topik-topik interview** | **Tema-tema untuk digali** |
| --- | --- |
| **Topik 1: Pegalaman test HIV testing selama kunjungan-kunjungan KIA.**  Pertanyaan: “*Bolehkan anda menceritakan pengalaman test HIV anda pada saat kunjungan-kunjungan KIA?”* | - Pengetahuan tentang HIV - Pengetahuan tentang PPIA - Interaksi petugas kesehatan-pasien - Alasan-alasan untuk menerima test HIV - Sejarah test HIV sebelumnya |
| **Topik 2: Pengalaman masuk dalam program PPIA.**  Pertanyaan*: “Bagaimana kisahnya anda mengetahui hasil test HIV anda?”* | - Waktu tunggu untuk hasil test - Durasi konseling - Kerahasiaan dan privasi - Alasan-alasan untuk masuk/tidak masuk dalam program PPIA - Sikap petugas kesehatan terhadap wanita sebelum dan sesudah terbukti HIV positif |
| **Topic 3: Pengalaman kelanjutan dalam PPIA.**  Pertanyaan: “*Apa yang mendorong anda untuk (tidak) lanjut dalam program?”* | - Fasilitator dan penghambat retensi dalam program PPIA: - Biaya transportasi - Jarak dengan fasilitas kesehatan - Stigma dan diskriminasi - Kebutuhan makanan - Keyakinan akan efikasi PPIA |
| **Topik 4: Saran-saran untuk meningkatkan program PPIA.**  Pertanyaan: “*Bagaimana menurut anda program PPIA dapat ditingkatkan?”* | - Strategi PPIA untuk wanita hamil dengan berbagai kondisi: - Kendala sosial (stigma dan diskriminasi) - Kendala geografis - Kendala finansial - Isu-isu lainnya yang disebutkan oleh wanita |
| **Topik 6: Penutup**  Pertanyaan: *“Adakah hal lainnya yang ingin anda tambahkan?”* |  |
