# Supplementary material for: Understanding women's uptake and adherence in Option B+ for prevention of mother-to-child HIV transmission in Papua, Indonesia: A qualitative study
Source: PLoS One. 2018 Jun 18;13(6):e0198329. doi: 10.1371/journal.pone.0198329 (PMC6005458; doi:10.1371/journal.pone.0198329)
Supplement: S4 File — (DOCX) [file pone.0198329.s004.docx]

#
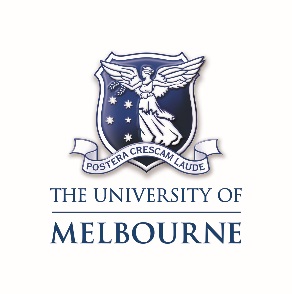
Interview Guide

## Centre for Health Policy

## ***Project: Understanding women’s uptake and adherence in Option B+ for prevention of mother-to-child HIV transmission in Papua, Indonesia: A qualitative study***

**Interview process:** The student researcher will conduct one on one in-depth interviews with health workers. The student researcher will explore each theme provided by participants and ask additional questions for missing or undetected themes before continuing to the next topic.

1. **Demographic information**
2. Age: _________(years)
3. Ethnic background: ____________
4. Religion:______________
5. Educational background:
6. Did not complete primary school
7. Primary school
8. Junior high school
9. Senior high school
10. College/University
11. Occupation:
12. Medical doctor
13. Midwife
14. Nurse
15. Counsellor

Logistic staff

1. Marital status:
2. Single
3. Married
4. Divorced
5. Widow(er)
6. Length of involvement in PMTCT program: ________(months)_______(weeks)
7. Lists of PMTCT training(s) received and year(s): _______________________________________________________________________________________________________________________________________________________________________________

| **III. Main Questions** | **Themes to explore**: |
| --- | --- |
| **Topic 1: Roles and responsibilities in PMTCT program.**  Question: “*Can you tell me about what you do in PMTCT program?”* | - Roles and responsibilities - Other roles and responsibilities at the hospital |
| **Topic 2: Management of pregnant women visiting ANC clinic for the first time.**  Question: “*What happens when a pregnant woman come to the clinic for the first time?”* | - PITC (provider-initiated testing and counselling) vs VCT (voluntary counselling and testing) - Patient flow system for PMTCT - PMTCT guideline adopted - Pre-test counselling: - Counselling content - Methods to verify women’s understanding - Estimate duration of counselling - Factors affecting the duration - Confidentiality and privacy |
| **Topic 3: Enrolment of women in PMTCT program.**  Question: “*What are the procedures taken when a pregnant woman requires a PMTCT program?”* | - Post-test counselling: - Counselling content - Methods to verify understanding - Estimate duration of counselling - Factors affecting the duration - Confidentiality and privacy - Reasons for enrolment/not enrolment in PMTCT program |
| **Topic 4: Retention of women in PMTCT program.**  Question: “*What kinds of measures are available to ensure women’s retention in the program?”* | - Facilitators and barriers to retention in PMTCT program: - Referral system - Community outreach - Commitment to the program - Stigma and discrimination - Logistic issues |
| **Topic 5: Suggestions to improve PMTCT program.**  Question: “*How do you think the current PMTCT program could be improved?”* | - PMTCT strategies for pregnant women under various circumstances: - Social constraints - Geographical constraints - Financial constraints |
| **Topic 6: Closing**  Question: *“Is there anything more you would like to add?”* |  |
