# Supplementary material for: Understanding women's uptake and adherence in Option B+ for prevention of mother-to-child HIV transmission in Papua, Indonesia: A qualitative study
Source: PLoS One. 2018 Jun 18;13(6):e0198329. doi: 10.1371/journal.pone.0198329 (PMC6005458; doi:10.1371/journal.pone.0198329)
Supplement: S5 File — (DOCX) [file pone.0198329.s005.docx]

#
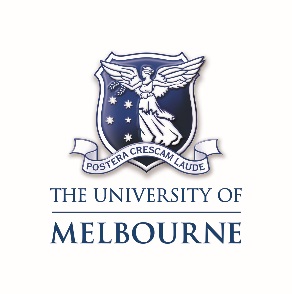
Panduan Interview

## Pusat Kebijakan Kesehatan

## ***Proyek: Memahami penerimaan dan kepatuhan wanita dalam Opsi B+ untuk pencegahan transmisi HIV dari ibu ke anak di Papua, Indonesia: Sebuah studi kualitatif***

**Proses interview:** Mahasiswa peneliti akan melakukan wawancara mendalam satu persatu dengan petugas kesehatan. Mahasiswa peneliti akan menggali setiap tema yang disebutkan oleh peserta dan mengajukan pertanyaan tambahan untuk tema yang hilang atau tidak disebutkan sebelum melanjutkan ke topik berikutnya.

1. **Informasi demografis**
2. Usia: _________(tahun)
3. Latarbelakang etnis: ____________
4. Agama:______________
5. Latarbelakang pendidikan:
6. Tidak menyelesaikan sekolah dasar
7. Sekolah dasar
8. Sekolah menengah pertama
9. Sekolah menengah atas
10. Universitas
11. Pekerjaan:
12. Dokter
13. Bidan
14. Perawat
15. Konselor
16. Petugas logistik
17. Status pernikahan:
18. Tidak menikah
19. Menikah
20. Cerai
21. Janda/duda
22. Lama keterlibatan dalam program PPIA: ________(bulan)_______(minggu)
23. Daftar pelatihan-pelatihan PPIA yang diterima dan tahun-tahunnya: _______________________________________________________________________________________________________________________________________________________________________________

| **III. Pertanyaan Utama** | **Tema-tema untuk digali** |
| --- | --- |
| **Topik 1: Peran dan tanggungjawab dalam program PPIA.**  Pertanyaan: “*Dapatkah anda menceritakan tentang apa yang anda lakukan dalam program PPIA?”* | - Tugas dan tanggungjawab - Tugas dan tanggungjawab lainnya di rumah sakit |
| **Topik 2: Manajemen ibu hamil yang mengunjungi klinik KIA untuk pertama kalinya.**  Pertanyaan: “*Apa yang terjadi ketika seorang wanita hamil datang ke klinik untuk pertama kalinya?”* | - KTIP (konseling testing inisiatif petugas) vs KTS (konseling dan testing sukarela) - Alur pasien untuk PPIA - Panduan PPIA yang digunakan - Konseling pre-test: - Isi konseling - Metode untuk mencek pemahaman wanita - Perkiraan durasi konseling - Faktor-faktor yang mempengaruhi durasi tersebut - Kerahasiaan dan privasi |
| **Topik 3: Pendaftaran wanita dalam program PPIA.**  Pertanyaan: “*Bagaimana prosedurnya jika seorang wanita hamil membutuhkan program PPIA?”* | - Konseling post-test: - Isi konseling - Metode untuk mencek pemahaman wanita - Perkiraan durasi konseling - Faktor-faktor yang mempengaruhi durasi tersebut - Kerahasiaan dan privasi - Penyebab-penyebab masuk/tidak masuk dalam program PPIA |
| **Topik 4: Retensi wanita dalam program PPIA.**  Pertanyaan: “*Jenis tindakan apa yang tersedia untuk memastikan retensi perempuan dalam program?”* | - Fasilitator dan penghambat retensi dalam program PPIA: - Sistem rujukan - Penjangkauan komunitas - Komitmen terhadap program - Stigma dan diskriminasi - Isu-isu logistik |
| **Topik 5: saran-saran untuk meningkatkan program PPIA.**  Pertanyaan: “*Bagaimana* *menurut anda program PPIA saat ini dapat ditingkatkan?”* | - Strategi-strategi PPIA untuk wanita hamil dengan berbagai kondisi: - Kendala sosial - Kendala geografis - Kendala finansial |
| **Topik 6: Penutup**  Pertanyaan: *“Adakah hal lainnya yang ingin anda tambahkan?”* |  |
